# Engineered artificial antigen presenting cells facilitate direct and efficient expansion of tumor infiltrating lymphocytes

**DOI:** 10.1186/1479-5876-9-131

**Published:** 2011-08-09

**Authors:** Qunrui Ye, Maria Loisiou, Bruce L Levine, Megan M Suhoski, James L Riley, Carl H June, George Coukos, Daniel J Powell

**Affiliations:** 1 Ovarian Cancer Research Center, Department of Obstetrics and Gynecology, Perelman School of Medicine, University of Pennsylvania, Philadelphia, PA, USA; 2 Abramson Family Cancer Research Institute, Department of Pathology and Laboratory Medicine, Perelman School of Medicine, University of Pennsylvania, Philadelphia, PA, USA; 3 Department of Pathology, Stanford School of Medicine, Stanford, CA, USA

## Abstract

**Background:**

Development of a standardized platform for the rapid expansion of tumor-infiltrating lymphocytes (TILs) with anti-tumor function from patients with limited TIL numbers or tumor tissues challenges their clinical application.

**Methods:**

To facilitate adoptive immunotherapy, we applied genetically-engineered K562 cell-based artificial antigen presenting cells (aAPCs) for the direct and rapid expansion of TILs isolated from primary cancer specimens.

**Results:**

TILs outgrown in IL-2 undergo rapid, CD28-independent expansion in response to aAPC stimulation that requires provision of exogenous IL-2 cytokine support. aAPCs induce numerical expansion of TILs that is statistically similar to an established rapid expansion method at a 100-fold lower feeder cell to TIL ratio, and greater than those achievable using anti-CD3/CD28 activation beads or extended IL-2 culture. aAPC-expanded TILs undergo numerical expansion of tumor antigen-specific cells, remain amenable to secondary aAPC-based expansion, and have low CD4/CD8 ratios and FOXP3+ CD4+ cell frequencies. TILs can also be expanded directly from fresh enzyme-digested tumor specimens when pulsed with aAPCs. These "young" TILs are tumor-reactive, positively skewed in CD8+ lymphocyte composition, CD28 and CD27 expression, and contain fewer FOXP3+ T cells compared to parallel IL-2 cultures.

**Conclusion:**

Genetically-enhanced aAPCs represent a standardized, "off-the-shelf" platform for the direct ex vivo expansion of TILs of suitable number, phenotype and function for use in adoptive immunotherapy.

## Introduction

Adoptive immunotherapy using tumor-reactive T lymphocytes has emerged as a powerful approach for the treatment of bulky, refractory cancer [1], however the ability to generate large numbers of TILs for therapy is a challenge that has significant regulatory hurdles, and requires technically sophisticated cell processing and extended in vitro lymphocyte culturing periods. Long-term culture of tumor-derived T cells in high-dose interleukin-2 (IL-2) allows for the generation of high numbers of TILs (>1 × 10^11^) but with preferential expansion of CD4+ lymphocytes [2-4]. Initial IL-2-based TIL expansion followed by a "rapid expansion method" (REM) [5-9] is a more time and labor efficient method, requiring an excess of irradiated allogeneic peripheral blood mononuclear cells (PBMC) as feeder cells, anti-CD3 antibody and high doses of IL-2, that can result in a 1,000-fold expansion of TILs over a 14-day period [9]. While routinely used, the REM has introduced technical, regulatory, and logistic challenges that have prevented larger and randomized clinical trials as a prelude to widespread application. First, large numbers of allogeneic feeders (200-fold excess), often from multiple donors, are required for clinical expansions. Second, allogeneic feeder cells harvested by large-volume leukapheresis from healthy donors exhibit donor to donor variability in their viability after cryopreservation and capacity to support TIL expansion, and thus test expansions are often required. Finally, this process necessitates additional extensive and costly laboratory testing of each individual donor cell product to confirm sterility.

Artificial antigen presenting cells (aAPCs) expressing ligands for the T cell receptor and costimulatory molecules can activate and expand T cells for transfer, while improving their potency and function. The first generation of aAPC consisted of anti-CD3 and anti-CD28 monoclonal antibodies (mAbs) covalently bound to magnetic beads (CD3/CD28 beads) which crosslink CD3 and CD28 on T cells, enabling efficient polyclonal expansion of circulating T cells (50 to 1000-fold) over 10-14 days of ex vivo culture with preferential expansion of naïve and memory CD4+ T cells [10], however their efficiency in TIL expansion has not been examined. Second generation cell-based aAPCs can substitute for natural APCs, mediate efficient expansion of antigen-specific T cells from peripheral blood [11-16] and stably express multiple gene inserts, including CD64 (the high-affinity Fc receptor), CD32 (the low-affinity Fc receptor), and CD137L (4-1BBL), among others [13,15]. Compared to beads, cell-based aAPCs bearing the costimulatory ligand CD137L can more efficiently induce the proliferation of antigen-experienced CD8+ CD28^- ^T cells from peripheral blood and improve their in vivo persistence and antitumor activity upon adoptive transfer to tumor-bearing mice [15,17]. In these studies, enhanced proliferation of antigen-experienced CD8+ CD28^- ^T cells mediated by aAPCs is dependent on CD137 ligation [15,17].

Unlike peripheral blood lymphocytes (PBL), most tumor antigen-specific CD8+ TILs derived from solid tumors express low levels of CD28 [18,19]. Together, the above studies suggest that approaches utilizing CD137 ligation may support ex vivo TIL expansion. In a trial of adoptive TIL transfer with REM generated cells, the persistence of TILs in vivo after infusion represented a major limitation to successful therapy [20]. In vivo persistence and clinical response were both associated with expression of the costimulatory molecules CD28 and CD27 by TILs, as well as their telomere length [18,21-24]. The REM requires extended duration TIL culture which results in telomere length shortening and reduced expression of CD28 and CD27 [18,25], thus there remains a need for the development of improved, standardized methods and materials for generating TILs rapidly for adoptive transfer with greater potency and engraftment capability.

Here we investigate the use of engineered K562 cell-based aAPCs as an "off-the-shelf" platform for ex vivo TIL expansion. K562 aAPCs that express CD137L offer the potential to expand antigen-experienced TILs and represent a potential new cell-based platform for the standardization of ex vivo TIL expansion. Ovarian cancer and melanoma biospecimens were used to test the notion that aAPC can stimulate TIL expansion in different tumor histotypes [26,27], based on the knowledge that TILs from these cancers can recognize autologous tumor as well as known tumor antigens in vitro [28-32], and exhibit tumor-specific reactivity ex vivo [33,34] and in vivo [5,7,35]. We found that aAPCs efficiently expand IL-2 cultured TILs from solid tumor specimens of ovarian cancer similar to the REM, resulting in a favorable CD4/8 T cell ratio, and low FOXP3+ CD4 T cell composition. aAPC-based TIL expansion depends on the provision of exogenous IL-2 cytokine support in culture and is largely CD28-independent. Under these conditions, tumor antigen-specific TILs with demonstrated anti-tumor reactivity can be expanded. Further, aAPC can induce the rapid and efficient expansion of TILs directly from freshly digested tumor samples, reducing overall culture time, and output TILs are highly skewed in CD8+ lymphocyte composition, possess high levels of CD28 and CD27 expression after activation and are amenable to secondary aAPC-based expansion. The aAPC platform as described here thus establishes a standardized methodology for the rapid, clinical-grade expansion of TILs for therapy.

## Materials and methods

### Generation of TILs

Patients were entered into an Institutional Review Board-approved clinical protocol and signed an informed consent prior to initiation of lymphocyte cultures. Generation of TILs was performed as described elsewhere [9]. Briefly, 2 mm^3 ^tumor fragments were cultured in complete media (CM) comprised of AIM-V medium (Invitrogen Life Technologies, Carlsbad, CA) supplemented with 2 mM glutamine (Mediatech, Inc. Manassas, VA), 100 U/ml penicillin (Invitrogen Life Technologies), 100 μg/ml streptomycin (Invitrogen Life Technologies), 5% heat-inactivated human AB serum (Valley Biomedical, Inc. Winchester, VA) and 600 IU/mL rhIL-2 (Chiron, Emeryville, CA). TILs established from fragments were grown for 3-4 weeks in CM and expanded fresh or cryopreserved in heat-inactivated HAB serum with 10% DMSO and stored at -180°C until the time of study. Tumor associated lymphocytes (TAL) obtained from ascites collections were seeded at 3e6 cells/well of a 24 well plate in CM. TIL growth was inspected about every other day using a low-power inverted microscope. Each initial well was considered to be an independent TIL culture and was maintained accordingly. For enzymatic digestion of solid tumors, tumor specimen was diced into RPMI-1640, washed and centrifuged at 800 rpm for 5 minutes at 15-22°C, and resuspended in enzymatic digestion buffer (0.2 mg/ml Collagenase and 30 units/ml of DNase in RPMI-1640) followed by overnight rotation at room temperature.

### aAPC preparation

KT64/BBL and KT32/BBL aAPCs were generated, cultured and prepared for co-culture as previously described [13,15]. Briefly, Fc-binding receptors on KT64/BBL aAPCs were pre-cleared of serum immunoglobulins by culture in serum free AIM-V medium (SFM) overnight and then irradiated at 10,000 rad. Anti-CD3 (OKT-3) with or without anti-CD28 (clone 9.3) mAbs were loaded on aAPCs at 0.5 ug/10^6 ^cells at 4°C for 30 minutes. Before use, aAPCs were washed twice with SFM. For KT32/BBL aAPCs, anti-CD3 and anti-CD28 antibodies were not washed out of culture medium, per established protocol [13,15]. For expansion of IL-2 cultured TILs, an optimal 2:1 aAPC to TIL ratio was established and used in all experiments.

### Expansion of TILs and TALs in vitro using aAPCs

10^6 ^heterogonous TILs or TALs were co-cultured with KT64/BBL or KT32/BBL aAPCs loaded with anti-CD3 with or without anti-CD28 antibody in one well of a 24 well plate. rhIL-2 (100 IU/ml) was added into co-cultures at day 2. Every other day the cell number was counted by on a Coulter Multisizer and adjusted to a concentration of 0.5-1 × 10^6 ^cells/ml until day 8. Expanding cocultures were transferred into an appropriately sized flask and suspended in CM containing rhIL-2 100 IU/ml depending on total cell numbers. Confirmatory hemacytometer counts including Trypan Blue exclusion were performed. After day 9, phenotypes of expanded TILs or TALs were examined by flow cytometry. Final expanded products were uniformly comprised by CD3+ TILs, TALs or PBLs, without aAPC contamination, as verified by cell sizing, morphology and flow cytometry. The total duration of cell expansion culture was between 9 and 14 days. At the end of culture, all remaining cells were frozen in 90% HAB serum and 10% DMSO for continued analysis. For comparison to other methods of T cell expansion, TILs or TALs were cultured in three conditions: with rhIL-2 (600 IU/ml) in CM; with anti-CD3/CD28 magnetic beads (3:1 beads to T cells) in rhIL-2 (100 IU/ml) (Chiron); or in a "rapid expansion method" condition (200:1 allogeneic PBMC:TILs, 30 ng/ml of OKT-3 anti-CD3 mAb and 6000 IU/ml rhIL-2 in 20 mL of CM in a T75 flask). For stimulation of fresh tumor digests, 10^6 ^total cells from tumor digested products were stimulated using an equivalent number of irradiated aAPC loaded with anti-CD3 mAb in media supplemented with 100 IU/mL IL-2.

### Antibodies and flow cytometric immunofluorescence analysis

Antibodies against human CD3, CD4, CD8, CD16, CD25, CD32, CD64 and CD137 were purchased from BD Bioscience. 7-AAD antibody for viability staining was purchased from BD Bioscience (San Jose, CA). HER2:369-377 peptide (KIFGSLAFL) and MART-1:26-35(27L) peptide (ELAGIGILTV) containing HLA-A2010 tetramers were purchased from Beckman Coulter, Inc. (Brea, CA). Anti-FOXP3 antibody (clone 259D) was obtained from BioLegend (San Diego, CA). Fresh TILs or TALs were resuspended in FACS buffer consisting of PBS with 2% FBS (Gemini Bioproducts) at 10^7 ^cells/ml and blocked with 10% normal mouse Ig (Caltag Laboratories) for 10 min on ice. A total of 10^6 ^cells in 100 μl were stained with fluoro-chrome-conjugated mAbs at 4°C for 40 min in the dark. In some cases, cells were briefly stained with 7-AAD antibody for nonviable cell exclusion after washing twice and subsequently analyzed in a FACSCanto II (BD Biosciences). FOXP3 staining was performed using the eBioscience fixation and permeablization kits according to the manufacturer's instructions and cells stained with the anti-FOXP3 antibody from BioLegend. K562 aAPCs antibody loading was performed using anti-CD3 (OKT3) purchased from eBioscience (San Diego, CA) and anti-CD28 mAbs (clone 9.3). For cell division assays, TILs or PBLs were labeled with 128 nM of carboxyfluorescein succinimidyl ester (CFSE). CFSE labeled TILs or PBLs were expanded with aAPCs, CD3/28 beads, rhIL-2 (600 IU/ml) or REM as described above. At day 6, the cells were stained with anti-CD3, anti-CD4 and anti-CD8 and examined for CFSE division by FACS. Statistical significance of phenotypic differences was determined using paired two-tailed T-test.

### ELISA assay for T cell function

Stimulation of TILs by tumor cells was assessed by IFN-γ secretion. 1 × 10^5 ^TILs were cultured with 1 × 10^5 ^target cells in triplicate overnight in a 96 well U bottom plate in 200 uL of CM containing 5% heat-inactivated human AB serum. Supernatants were harvested and analyzed for IFN-γ by ELISA, according to manufacturer's instruction (Biolegend, San Diego, CA). Values represent the mean cytokine concentration (pg/mL) ± SD of triplicate wells.

## Results

### KT64/BBL aAPCs-based expansion TILs

K562 cells expressing CD64, CD137L and CD28 ligands CD80 and CD86, pulsed with anti-CD3 antibody efficiently activate and expand CD8+ CD28- T cells and antigen-specific T cells from peripheral blood when co-cultured at a 0.5:1 aAPC to T cell ratio in the absence of exogenous IL-2 and in a CD137L dependent manner [15]. We therefore hypothesized that tumor infiltrating lymphocytes (TILs) derived from cancer lesions could be efficiently expanded to therapeutic treatment numbers using a K562 cell-based aAPC platform. To generate cell-based aAPCs, the parental K562 cell line was engineered to stably co-express the high-affinity Fc receptor CD64 and the costimulatory ligand CD137L (4-1BBL) by lentiviral gene transduction. Single cell clones (referred to as KT64/BBL) were isolated by flow-sorting and their CD64 and CD137L surface expression was confirmed by flow cytometry (Additional file 1**Figure S1a**). KT64/BBL aAPCs were cultured in the absence of serum to pre-clear CD64 of serum derived immunoglobulins, irradiated and then loaded with anti-CD3 and anti-CD28 agonist monoclonal antibodies (mAbs) for TIL expansion.

**Figure 1:**
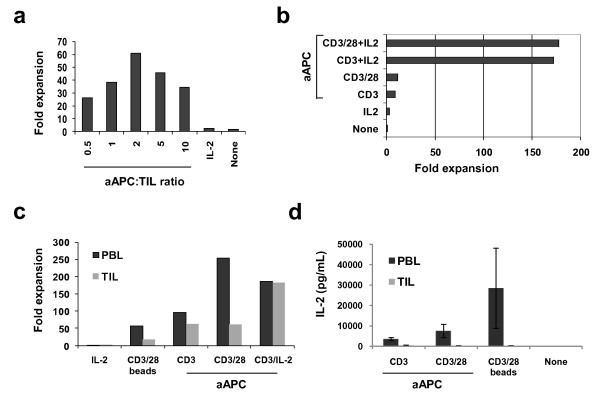
**KT64/BBL aAPCs support the expansion of TILs in a CD28-independent manner**. (**a**) TILs cultures established for 3-4 weeks in 600 IU/ml IL-2 were expanded using aAPCs loaded with anti-CD3 and anti-CD28 mAbs at various aAPC to T cell ratios in the continued presence of IL-2 (100 IU/mL). In this representative experiment (one of three), a 62-fold expansion of TILs was achieved 9 days after a single stimulation with aAPCs at the 2:1 aAPC to T cell ratio. A 3-fold expansion occurred after continued culture in IL-2. TILs stimulated with aAPCs underwent greater expansion at all aAPC to TIL ratios compared to continued growth in IL-2 or growth in medium alone. (**b**) KT64/BBL aAPC-based TIL expansion is CD28 costimulation-independent but augmented by provision of IL-2 support. Established TIL cultures were expanded for 9 days using aAPC loaded with anti-CD3 antibody in the presence or absence of clone 9.3 anti-CD28 antibody, in the presence or absence of IL-2 supplement. (**c**) CD28 costimulation augments the aAPC-based expansion of peripheral blood T cells, but not autologous TILs. CD3/28 beads do not support TIL expansion (3:1 bead to T cell ratio). Day 9 cell counts are shown. (**d**) TILs stimulated with KT64/BBL aAPCs with or without anti-CD28 antibody do not secrete IL-2 after overnight culture, but peripheral blood lymphocytes do. IL-2 secretion by PBL is increased by provision of CD28 costimulation and supported by CD3/28 bead stimulation. Mean IL-2 (pg/mL) concentration ± SEM from three independent TIL cultures is shown.

TIL cultures for expansion were outgrown from solid ovarian cancer fragments for 3-4 weeks in culture media (CM) containing 600 IU/mL rhIL-2 cytokine, as described [4,9], and were comprised of >95% CD3+ T cells and <1.5% NK cells. To test the capacity of antibody-loaded aAPCs to mediate ex vivo expansion of TILs, aAPC were co-cultured with TILs at aAPC to TIL ratios ranging between 0.5 and 10 to 1 in the continued presence of IL-2 (100 IU/ml). Peak TIL expansion was achieved at the 2:1 aAPC to T cell ratio (Figure 1a), which contrasts the 200:1 feeder to T cell ratio commonly used in REM-based TIL expansion [9]. The 2:1 aAPC to T cell ratio was therefore used for the experiments detailed below. The contribution of CD137L to TIL expansion was confirmed using control KT64 aAPCs lacking CD137L expression, which mediated diminished TIL expansion compared to KT64/BBL (Additional file 1**Figure S1a,b**), consistent with our prior study using antigen-experienced T cells [15]. Since our first generation of K562 based aAPC (referred to as KT32/BBL) relied upon the low affinity Fc receptor CD32 for anti-CD3 antibody loading and demonstrated the capacity to expand circulating T cells [15], we evaluated the relative efficiency of CD32 and CD64-expressing aAPCs for expanding TILs. KT64/BBL aAPCs were superior to KT32/BBL aAPCs, and therefore used in all further experiments (Additional file 2**Figure S2**).

### Robust expansion of TILs is dependent upon IL-2, but not CD28 costimulation

To investigate the impact of CD28 costimulation and IL-2 on aAPC-mediated TIL expansion, KT64/BBL aAPCs were loaded with anti-CD3 mAb +/- anti-CD28 mAb and used to stimulate TILs in the presence or absence of 100 IU/ml of IL-2 (Figure 1b). In the absence of IL-2, TILs underwent minimal expansion after stimulation with aAPCs loaded with anti-CD3 mAb with (11-fold) or without anti-CD28 mAb (9-fold), albeit more than when continually grown in IL-2 (3-fold). By comparison, addition of IL-2 to aAPC-based expansion induced vigorous numerical growth of TILs (>170-fold) in the presence or absence of anti-CD28 mAb, and the level of TIL expansion was similar whether or not anti-CD28 mAb was loaded onto the aAPCs. These results demonstrate that cell-based aAPC-mediated TIL expansion is largely independent of CD28 signaling when 4-1BBL is provided on aAPC, but dramatically improved by addition of IL-2 cytokine to culture.

The limited contribution provided by anti-CD28 mAb to the expansion of TILs in the absence of IL-2 counters that previously observed for peripheral blood T lymphocytes (PBLs) from healthy donors where CD28 costimulation in concert with TCR signaling induces robust proliferation [13,15]. We therefore evaluated the contribution of CD28 in the expansion of TILs and PBLs collected from the same patient with ovarian cancer. In paired comparison, measurement of CD28 expression on matched TILs and PBLs from the same patients revealed a higher relative expression of surface CD28 by T cells from the circulation than by T cells from tumor in all cases (Additional file 3**Figure S3**). Among CD3+ TILs, more CD4+ TILs expressed CD28 than CD8+ TILs (76.5 ± 32.9% vs. 34.7 ± 12.2%, respectively; p = 0.003). CD3+ T cells from the blood were heterogeneous in differentiation state and comprised of naïve (CD45RO- CD62L+), central memory (CD45RO+ CD62L+), and effector memory (CD45RO+ CD62L-) cell subsets; TILs however were comprised primarily of cells with a more differentiated, effector memory phenotype (representative examples are shown in Additional file 3**Figure S3**).

Consistent with their disparate differentiation phenotypes, peripheral blood T cells and TILs from the same patient demonstrated a relative difference in expansion in response to aAPC stimulation. The expansion of TILs in response to stimulation with aAPCs loaded with anti-CD3 mAb with or without CD28 agonist mAb co-loading was modest and similar (62-fold v. 63-fold, respectively), but was substantially augmented by the addition of IL-2 to culture (182-fold; Figure 1c). PBLs in parallel culture exhibited greater expansion in response to anti-CD3 mAb loaded aAPC stimulation compared to TIL, whether or not CD28 signaling was intact, however, PBL expansion was substantially elevated when the aAPCs were also loaded with CD28 agonist mAb (254-fold), relative to anti-CD3 mAb alone (95-fold). In the absence of CD28 costimulation, robust PBL expansion could be restored by addition of exogenous IL-2 cytokine (187-fold). Although PBL expansion in the condition of CD28 costimulation out-performed the addition of IL-2 at day 9 (Figure 1c), IL-2 supplementation was superior to CD28 costimulation by day 11 of PBL culture (737-fold v. 340-fold, respectively); at this time point, TIL cultures were unchanged in expansion hierarchy with a 287-fold expansion in the CD3/IL-2 condition. Consistent with previous findings[15], PBLs stimulated with anti-CD3 and anti-CD28 mAb loaded aAPCs expanded better than those stimulated with magnetic beads coated with anti-CD3 and CD28 mAbs to crosslink endogenous CD3 and CD28 (254-fold v. 56-fold, respectively; Figure 1c). TILs stimulated with CD3/CD28 beads did not undergo robust expansion (18-fold).

Supplement of TIL cultures with IL-2 cytokine, but not CD28 costimulation, during aAPC-induced stimulation dramatically improved TIL expansion, while PBLs showed improved expansion in response to aAPC with addition of either IL-2 or CD28 costimulation. This suggests that PBLs, which express elevated levels of CD28 relative to TILs, may produce and secrete more IL-2 when costimulated than their CD28^low ^TIL counterparts, thus supporting T cell expansion. Consistent with this notion, cytokine secretion analysis performed on supernatants from TILs or PBLs stimulated overnight with anti-CD3 mAb loaded aAPCs +/- anti-CD28 mAb revealed that TILs produce little to no IL-2 when stimulated with aAPC either with or without CD28 costimulation, or with CD3/CD28 beads (Figure 1d). By contrast, PBLs secreted high levels of IL-2 in response to aAPC which was augmented by the addition of CD28 agonist mAb loading. CD3/CD28 bead stimulation of PBLs resulted in an even greater level of IL-2 production than that achieved with aAPC. Both TILs and PBL secreted IFN-γ and TNF-α in response to aAPC and bead stimulation (not shown), indicating that the lack of IL-2 production by TILs was not a result of functional anergy.

### Comparison with conventional clinical expansion systems for TILs

**Figure 2:**
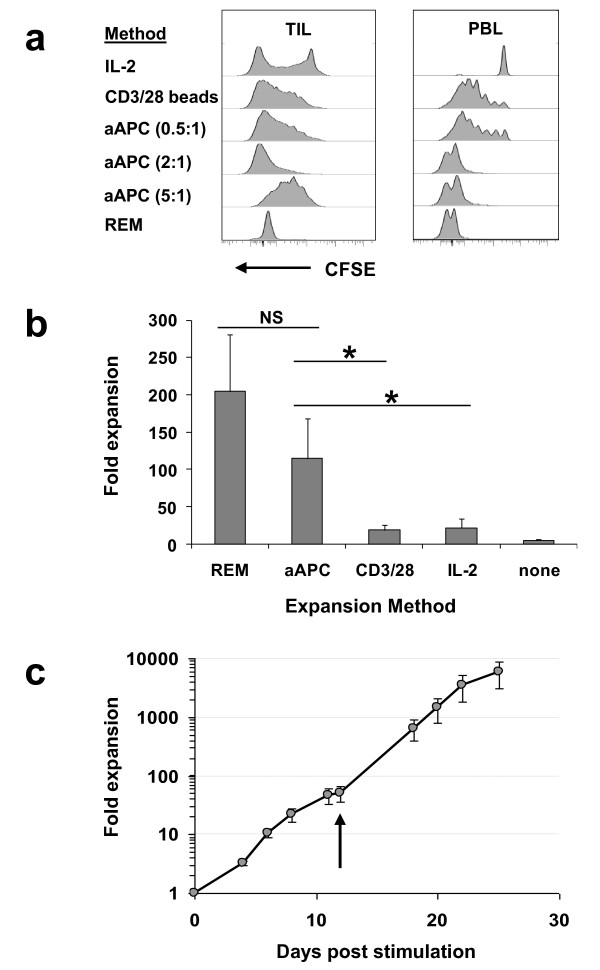
**A comparison of the KT64/BBL aAPC platform with previously established methods for TIL expansion**. (**a**) TILs undergo extensive cell division when stimulated with aAPC at the 2:1 aAPC to T cell ratio. TILs or peripheral blood T cells were labeled with CFSE and stimulated with aAPC at either 0.5, 2, or 5 to 1 ratios with TILs, REM, CD3/28 beads or 600 IU/mL IL-2. Cell division was measured using CFSE dilution by CD3+ T cells 6 days after stimulation. (**b**) TILs rapidly expand in response to aAPC or REM-based expansion. Seven different TIL cultures established in IL-2 were stimulated using either KT64/BBL aAPC loaded with anti-CD3 antibody and supplemented with 100 IU/mL IL-2 (aAPC); rapid expansion with anti-CD3 antibody, high-dose IL-2 (6000 IU/mL) and excess allogeneic feeder cells (REM); anti-CD3/28 antibody-coated beads stimulation at a 3:1 bead to TIL ratio (CD3/28); continued culture in 600 IU/mL IL-2 (IL-2); or culture medium alone. Results reflect the mean ± SEM day 9 viable cell counts for 6 independent expansions. (**c**) Robust secondary TIL expansion was achieved using the aAPC platform. Secondary TIL expansion was initiated 12 days after primary aAPC stimulation and cultured for an addition 13 days. Values represent the mean of three TIL expansion ± SEM. Arrow indicates the time of secondary stimulation.

To date, clinical preparation of TILs has largely relied upon expansion by IL-2 alone [4,36] and, more recently, by the "rapid expansion method" (REM) of anti-CD3 antibody, allogeneic feeder cells and IL-2 [5,8,9]. For polyclonal expansion of peripheral blood T lymphocytes, CD3/CD28 beads have been used [10], however their application for TIL expansion has not been reported. We compared the relative effectiveness of KT64/BBL aAPCs and other established culture methods of TIL expansion. TIL cultures outgrown in IL-2 containing CM and primary PBLs were either continually cultured in 600 IU/mL IL-2, or activated with CD3/CD28 beads, REM or KT64/BBL aAPCs. PBLs that were cultured in the presence of IL-2 did not divide, but underwent significant cell division in response to CD3/CD28 beads, although a fraction of cells remained undivided (Figure 2a). CD3/CD28 bead-induced cell division by PBLs was suboptimal and similar in level to that observed after activation with KT64/BBL aAPCs loaded anti-CD3 mAb at the 0.5:1 aAPC to T cell ratio. By comparison, all PBLs divided extensively after stimulation with aAPCs at aAPC to T cell ratios of 2:1 and 5:1, or after expansion by REM.

In contrast to PBLs, a portion of TILs underwent IL-2 induced cell division, likely due to their pre-conditioning in IL-2; however a substantial number of TILs in these cultures did not divide. TILs cultured with aAPCs at the 2:1 ratio underwent extensive cell division, which was similar to that observed in TILs stimulated by the REM, and consistent with T cell counts (Figure 2a). Nearly all TILs stimulated with CD3/CD28 beads or aAPCs at the 0.5:1 ratio divided, albeit at a moderate level. At the 5:1 ratio, most TILs had undergone an intermediate level of cell division, consistent with cell counts (Figure 1b), likely resulting from overcrowding due to space limitations in culture vessels. After 9 days of culture, TILs stimulated by REM or KT64/BBL aAPCs had undergone significant cell expansion, relative to continued IL-2 culture (p < 0.05 by paired t-test; Figure 2b). TILs underwent a mean fold expansion of 205 ± 77 (mean ± SEM) when stimulated with the REM, and a 114 ± 54 fold-expansion by aAPC, a difference which was not statistically significant (p = 0.15). Expansion of TILs with CD3/CD28 beads was not robust, resulting in an 18.8 ± 7.3 mean fold expansion, and was not significantly different from continuous IL-2 culture (21.8 ± 11.9-fold, p = 0.32) or media alone control (4.8 ± 2.2-fold; p = 0.12). To evaluate their continued expansion potential, TILs that had expanded less than 100-fold after a single-round of aAPC stimulation were restimulated with aAPC. After restimulation, TILs underwent further robust expansion, reaching 10,000-fold growth over 25 days (Figure 2c).

### TIL phenotype following aAPC expansion

**Figure 3:**
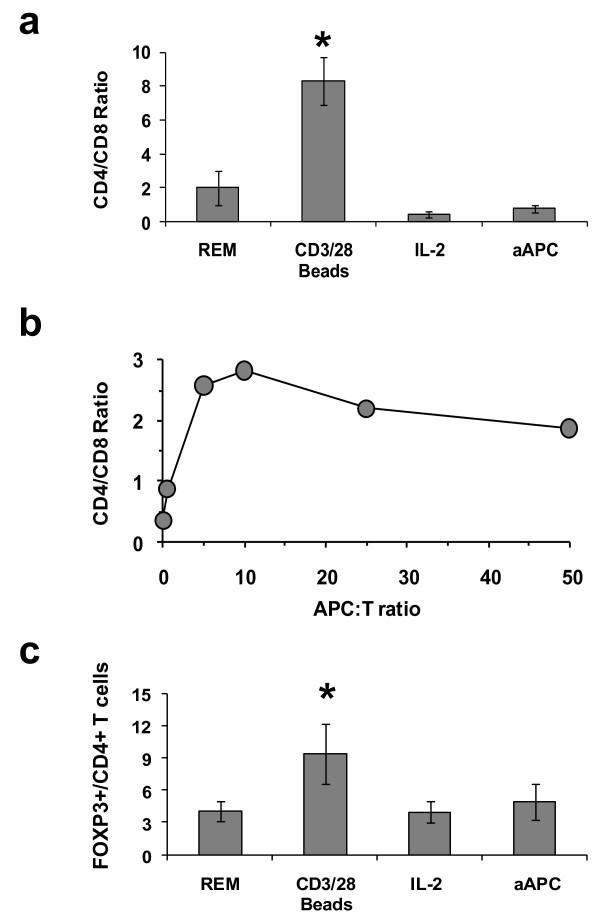
**TILs expanded with KT64/BBL aAPCs are comprised of favorable T cell subsets**. (**a**) TILs expanded with aAPC are preferentially comprised of CD8+ T cells. TILs or TALs expanded for 9-11 days under conditions of REM, CD3/28 beads, continued IL-2 growth (600 IU/mL) or aAPC were evaluated for CD4 and CD8 T cells composition. All expanded TIL or TAL cultures were uniformly comprised of CD3+ T cells. Mean ± SEM of six independent expansions is shown. Asterisk indicates a statistically significant increase in CD4:CD8 ratio relative to all other conditions (p < 0.04). (**b**) Higher CD4: CD8 T cell ratios are observed with increased aAPC: TIL ratios. The result of a representative TIL expansion experiment is shown. (**c**) FOXP3+ CD4 T cell frequencies are low following aAPC-based expansion. TILs or TALs stimulated and cultured under various conditions for 9-11 days were stained for CD3, CD4 and FOXP3. At day 9-11 post stimulation, TILs had returned to resting TIL cell size. Mean ± SEM of six independent expansions is shown. Asterisk indicates a statistically significant increase in FOXP3+ CD4 T cell frequency relative to all other conditions (p < 0.05).

Flow cytometric analysis was performed to determine the impact of expansion by the various methods on TIL phenotype. Prior to stimulation, CD4 T cells dominated TIL cultures at a CD4: CD8 ratio of 2.05 ± 0.30 (mean ± SEM; n = 6). After expansion, aAPC stimulated TILs had a low CD4:CD8 T cell ratio (0.77 ± 0.21) that was statistically similar to that observed after REM or IL-2 based expansion (Figure 3a). TILs stimulated with CD3/CD28 beads were largely comprised of CD4 T cells with a CD4:CD8 ratio that was higher than those observed in all other conditions (p < 0.04), likely due to the CD8+ TIL subset containing a much higher proportion of CD28- cells than the CD4+ subset. Although a favorable CD4:CD8 ratio (<1) was seen at the 2:1 aAPC:TIL ratio, higher aAPC:TIL ratios resulted in increased CD4:CD8 ratios following stimulation and culture (Figure 3b). CD16+ NK cells, which were detectable at levels <1.5% of starting IL-2 cultured TIL samples, were not detectable after aAPC-based expansion (not shown). Among CD4+ T cells in TIL cultures, the frequency of FOXP3+ CD4+ T cells was highest in TILs that had been expanded with CD3/CD28 beads, which was significantly greater than in TILs expanded with aAPC (p < 0.05; Figure 3c). Since in vitro activation of T cells can induce transient FOXP3 upregulation [37], analysis was performed only after TILs had rested down as defined by a return of cells to their pre-expansion size, measured using a Multisizer 3 Cell Sizing device, and a lack of spontaneous proinflammaorty cytokine release. The level of FOXP3+ CD4+ T cells was similar among TILs expanded with aAPC, REM or continuous IL-2 culture. The differentiation phenotype of TILs after expansion was not significantly different when stimulated with KT64/BBL, beads, REM or IL-2 with a predominant CD28^int ^CD27^low ^CD45RA^neg ^CD45RO^pos ^CCR7^low ^CD62L^int ^phenotype (not shown).

### Maintenance of tumor antigen-specific T cells after aAPC-based expansion

**Figure 4:**
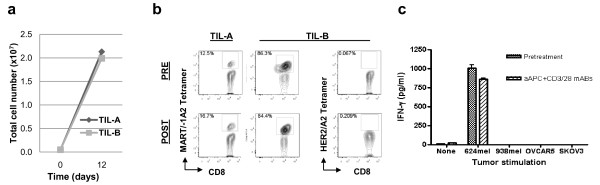
**Numerical expansion of tumor antigen-specific T cells using KT64/BBL aAPC**. (**a**) Melanoma TIL that had been outgrown in for 4 weeks in IL-2 expand rapidly using KT64/BBL aAPC loaded with anti-CD3/28 in the presence of IL-2 (100 IU/mL). Day 9 expansion results for representative samples (TIL-A and TIL-B) are shown. (**b**) MART-1 peptide-specific CD8+ T cells are detectable in pre- and post-expansion TILs via flow cytometry using MART-1:27L-35 peptide/HLA-A*0201 tetramers. TILs were stained for viability, CD3, CD8 and MART-1:27L-35 peptide/HLA-A0201 tetramers. Viable CD3+ T cell gating was performed. (**c**) aAPC expanded melanoma TILs retain HLA-restricted tumor reactivity in a standard co-culture and cytokine detection assay. 10^5 ^aAPC-expanded TILs were co-cultured with 10^5 ^624 (A2+ MART-1+) or 938 (A2- MART-1+) melanoma cells, or OVCAR5 (A2+ MART-1-) or SKOV3 (A2- MART-1-) ovarian cancer cells. After overnight culture, supernatants were measured for secreted IFN-g.

TILs outgrown from ovarian cancer can recognize and respond to stimulation with autologous tumor as well as known tumor antigens ex vivo [28-34], although the prevalence of tumor-reactive TILs in ovarian cancer is low. To evaluate whether TILs with specific tumor reactivity are maintained in aAPC expanded cultures, we selected TILs isolated and expanded in IL-2 from melanoma fragments, where tumor antigen-specific T cells are frequently detected for expansion with KT64/BBL aAPCs (Figure 4a). More than a 220-fold expansion was observed over 10-12 days culture in independent assays. MART-1:27-35 peptide-specific CD8+ TILs from HLA-A2+ patients, which were readily detected in pretreatment TILs, were also observed post-expanded TIL populations (Figure 4b). Control HER2:369-377 tetramer staining was negative in these melanoma TIL cultures. In co-culture assays, aAPC-expanded TILs containing MART-1-specific CD8+ T cells retained the ability to recognize and respond to the HLA-matched, MART-1 expressing melanoma cells line 624, but not when stimulated with HLA-A2^neg ^MART-1^+ ^melanoma (938), or HLA-matched MART-1^neg ^(OVCAR5) or HLA-A2^neg ^MART-1^neg ^(SKOV3) ovarian cancer cell lines (Figure 4c), indicating maintenance of anti-tumor reactivity by aAPC expanded TILs.

### Direct expansion of TILs from fresh digested tumor specimens

**Figure 5:**
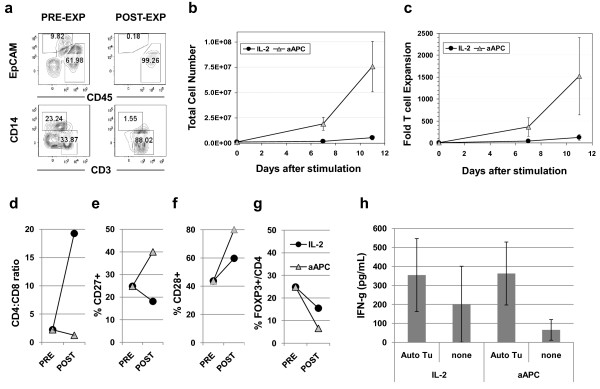
**Young TILs with favorable cell subset composition can be expanded directly from fresh tumor digests using aAPCs**. (**a**) Fresh ovarian cancer digests (PRE-EXP) are comprised by a heterogenous mix of EpCAM+ tumor cells and CD45+ leukocytes, containing CD14+ monocytes and CD3+ T cells; aAPC expanded digests (POST-EXP) contain only CD45+ CD3+ T cells. Lower dot plots are CD45+ gated. (**b**) 10^6 ^total tumor digest cells were stimulated with 10^6 ^aAPC loaded with anti-CD3 and anti-CD28 agonist antibody in CM containing 100 IU/mL IL-2, or cultured in 600 IU/mL IL-2 alone. Mean viable cell counts ± SEM are shown (n = 7). (**c**) Fold expansion of CD3+ TILs. Calculated viable absolute T cell numbers are shown (Total T cell number times % viable CD3+). (**d**) Ratio of CD4 + TILs to CD8+ TILs pre- and post-expansion with either aAPC or IL-2 alone; (**e**) percentage of CD3+ TILs expressing CD27; (**f**) or CD28; (**g**) percentage of CD4+ CD3+ TILs expressing FOXP3. Values in (d-g) represent the mean expression of the indicated molecule by 4 independently expanded TILs. (**h**) TILs expanded directly from enzyme-digested tumor specimens using KT64/BBL aAPC demonstrated autologous tumor reactivity. 10^5 ^aAPC-expanded TILs or 10^5 ^TILs outgrown in 600 IU/mL of IL-2 were co-cultured overnight with 10^5 ^autologous tumor cells or not stimulated (none). Anti-CD3/28 bead stimulation was applied as positive control. Mean concentration of IFN-g (pg/mL ± SEM) detected in supernatants from paired aAPC- and IL-2-expanded TIL cultures from 4 independent ovarian cancer specimens with anti-tumor reactivity is shown.

Extended culture of human T cells results in progressive T cell differentiation and loss of replicative potential which impairs in vivo T cell persistence and anti-tumor responses following adoptive cell transfer [20,24,25,38]. We therefore tested whether so-called "young" TILs could be generated via direct aAPC-based expansion of TILs. We modified the approach of TIL generation, using primary co-cultures of collagenase-digested tumor specimens rather than IL-2 outgrown microcultures derived from solid tumor fragments. Following enzymatic digestion, tumor specimens were comprised of EpCAM+ tumors cells, and a CD45+ leukocyte population that contained CD14+ monocytes and CD3+ T cells, as well as a CD14- CD3- leukocyte subset (Figure 5a). The frequency of CD3+ T cells in the starting digested tumor specimens was low, ranging from 0.76% to 15.68% of all viable cells (mean 6.3 ± 2.1%, n = 7). Stimulation of 1 million total cells from tumor digested products with an equivalent number of irradiated aAPC loaded with anti-CD3/28 antibodies in media supplemented with IL-2 yielded on average a 75-fold numerical expansion of total cells after 11 days, which was substantially higher than that achieved by IL-2 culture alone (mean of 5.6-fold; Figure 5b). Stimulation of the heterogenous tumor cell product resulted in the rapid, preferential expansion of CD3+ CD45+ T cells, which dominated the final cell product (Figure 5a). CD64+ CD137+ aAPCs were not detectable in the final TIL preparation and no viable aAPCs were observed in independent parallel cultures of aAPC alone after day six. Longitudinal enumeration of CD3+ TILs during expansion revealed that TILs, which were a relatively small portion of the starting digested tumor cell product, underwent a robust 1,500-fold mean expansion over 11 days in culture (Figure 5c). "Young" TILs that expanded to modest levels (185-fold mean expansion) were also amenable to secondary expansion with KT64/BBL aAPC, reaching an average total level of ~25,000-fold expansion 8 days after restimulation (Additional file 4**Figure S4**). Phenotypic analysis revealed that "young" TILs that had been expanded directly from solid tumor digests with aAPC trended toward having increased CD8+ T lymphocyte composition (Figure 5d), a higher frequency of T cells expressing the costimulatory molecules CD27 and CD28 (Figure 5e,f), and reduced frequencies of CD4+ T cells expressing FOXP3, relative to TILs cultured in IL-2 in parallel (Figure 5g), although not to the level of statistical significance. Young ovarian TILs that had been expanded directly from fresh enzyme-digested tumor specimens exhibited autologous tumor reactivity ex vivo (IFN-g secretion >200 pg/mL and twice background) that was statistically similar to the reactivity of TILs that had been outgrown in parallel IL-2 cultures (p = 0.95; n = 4; Figure 5h). Reactivity to MHC-mismatched ovarian cancer cell lines was not observed (not shown) Thus, TILs can be vigorously expanded directly from enzyme-digested tumor specimens ex vivo with KT64/BBL aAPCs, and display favorable phenotypic and functional attributes for the application of adoptive immunotherapy of cancer.

## Discussion

TIL-based therapy for cancer has shown significant promise in the clinic [5-7,35,39,40] but TIL expansion procedures require significant simplification to allow for wider application, improved cell product development and better patient outcomes. The results of this study demonstrate the novel applicability of a more efficient cellular aAPC-based platform for expansion of human T lymphocytes derived from solid tumor explants than has previously been reported. The engineered KT64/BBL aAPC line evaluated in this study represents an attractive "off-the-shelf" platform for ex vivo TIL expansion since aAPC (i) can be grown to large number and cryopreserved for the establishment of master and working cell banks, thus meeting the needs of even the largest cell cultures, (ii) reduce sample variability, preparative time requirements and regulatory issues that surround the use of donor PBMCs as a feeder cell source, (iii) are amenable to further genetic engineering or antibody loading to broaden or fine-tune the spectrum of costimulatory or adhesion molecules expressed, (iv) lack endogenous MHC expression thus eliminating issues of HLA-compatibility, and (v) alleviate possible infectious agent concerns related to the use of donor PBMC as feeder cells.

TILs, which generally express lower levels of CD28 than blood-derived T cells, efficiently expand using aAPCs in a CD28 independent manner, but require the addition of exogenous IL-2, likely due to the inability to TILs to produce their own IL-2 when stimulated with or with or without CD28 costimulation. The level of TIL expansion achieved using aAPC is similar to that attained by the REM, adapted from Riddell [8,9], and far exceeds that of continued culturing in IL-2 or stimulation with beads coated with anti-CD3 and anti-CD28 mAb. The expansion levels reached over 9-11 days of culture using REM and aAPC as performed here in small-scale using extended IL-2 cultured ovarian TILs is less than those levels achieved elsewhere with melanoma TILs over 14 days by REM [9]. These differences may be a reflection of dissimilar culture duration, scale, feeder cell capacity or tumor type. Compared to the 200:1 feeder to TIL ratio and 6000 IU/mL IL-2 used for the REM, stimulation of TILs with aAPCs at a 2:1 ratio and 100 IU/mL IL-2 efficiently expand TILs. More so, aAPC-expanded cells remain sensitive to secondary aAPC-based re-stimulation, allowing for a nearly 10,000-fold total cell expansion. aAPC-expanded TILs are skewed in CD8+ T cell contribution with few FOXP3+ cells among the smaller CD4+ T cell population, which may benefit adoptive cell transfer protocols [41,42]. Importantly, the aAPC platform supports the numerical expansion of tumor antigen-specific T cells within the TIL population. This likely reflects the use of TIL cultures established from tumor fragments or digests over 3-4 weeks in IL-2, which has been shown to promote TIL differentiation, telomere shortening and senescence [25].

Adoptive transfer of TILs possessing properties of less differentiated T cells, such as high surface expression of the costimulatory molecules CD28 and CD27 and long telomeres (>5 kb), is associated with their increased persistence in vivo and correlates with objective cancer regression [18,20,22-24]. Modification of TIL culture conditions, including shortening the duration of culture, use of alternative common γ-chain signaling cytokines and cytokine concentration [25,43,44], can skew TIL differentiation status in vitro and improve their in vivo potency. Alternatively, enrichment for particular T cell subsets, such as cytotoxic CD8+ T cells, may improve overall TIL potency and function [42]. We demonstrate that TILs stimulated with aAPCs directly from fresh tumor digests undergo more robust expansion, have increased CD8+ T cell composition, contain a greater numbers of cells expressing CD28 and CD27, and have similar function compared to parallel TILs developed under continuous IL-2 culture conditions. Under aAPC conditions, TILs selectively expand in culture, while tumor cells do not. Recent attempts at generating "young" TILs through minimal cell culture rely upon short-term (10-18 day) IL-2 incubation followed by REM expansion of about 14 days [25,35]. Our results extend upon these findings by demonstrating that even short term culturing in IL-2 alone can have a negative impact on overall TIL subset composition and differentiation phenotype. Direct TIL stimulation by aAPC minimizes overall culture time and the negative effects of extended in vitro population doubling. Minimized TIL expansion and culture as described here stands to reduce overall cell processing time and positively impact TIL subset and differentiation, which may facilitate wider application of TIL-based therapy and improve patient outcome. Based in part on these results, we have now also established and tested several Master and Working Cell Banks of K562 aAPCs. Biologics Master Files have been submitted to the FDA in preparation for use as ex vivo ancillary reagents in adoptive immunotherapy clinical trials.

## Conclusion

In this study, we show that cell-based aAPCs represent a stand-alone, standardized platform for rapid and efficient ex vivo expansion of tumor-infiltrating lymphocytes of sufficient number and quality for use in adoptive immunotherapy. aAPCs can be used to expand long-term, IL-2 cultured TIL cultures as well as generate less differentiated "young" TIL cultures with tumor-reactivity via direct expansion from enzyme-digested tumors. We conclude that aAPCs overcome costly technical, regulatory, and logistic challenges of allogeneic feeder cells, establishing aAPCs a preferable, standardized methodology for the rapid, clinical-grade expansion of TILs for therapy.

## Competing interests

The authors declare that they have no competing interests.

## Authors' contributions

QY carried out T cell expansions, cell analysis and data summary. ML carried out T cell expansions and cell analysis. BLL participated in designing the study and drafting the manuscript. MMS participated in T cell expansion. JLR participated in aAPC production and drafting the manuscript. CHJ participated in aAPC production and designing the study. GC participated in designing the study. DJP conceived, designed and coordinated the study and drafted the manuscript. All authors read and approved the final manuscript.

## Supplementary Material

Additional file 1 **Additional Figure S1**. Characteristics of KT64/BBL aAPCs used for TIL expansion. 4-1BBL expression by the aAPC has a positive impact on TIL expansion potential. **KT64/BBL aAPCs were generated to support the expansion of TILs**. (**a**) aAPCs were genetically engineered with recombinant lentiviruses to express CD64 and CD137 (4-1BBL; referred to as KT64/BBL) or CD64 alone (KT64). Engineered cells were isolated by flow-sorting. Enriched KT64/BBL cells expressed high levels of CD64 and CD137L whereas KT64 expressed high levels of CD64 but not CD137L, as measured by flow cytometry. Specific antibodies are shown in gray; isotype antibody control is shown in black. (**b**) TIL expansion is augmented by CD137L stimulation. KT64/BBL aAPC pulsed with anti-CD3 antibody (0.5 ug/10^6 ^cells) and anti-CD28 antibody (0.5 ug/10^6 ^cells) stimulated enhanced TIL expansion at a 2:1 aAPC to T cell ratio in the presence of exogenous IL-2 (100 IU/ml), compared to KT64 control aAPC under identical conditions. Click here for file

Additional file 2 **Additional Figure S2**. High affinity Fc gamma receptor CD64 is superior to the low affinity CD32 receptor for TIL expansion. **K562 aAPC engineered to express CD64, but not CD32, induce rapid TIL expansion**. K562 cells engineered to express 4-1BBL and the low affinity CD32/Fc-gammaRIII (KT32/BBL) or the high affinity CD64/FcgammaR1 receptor (KT64/BBL) were pulsed with anti-CD3 antibody (0.5 ug/10^6 ^cells) with or without anti-CD28 antibody (0.5 ug/10^6 ^cells) and used to stimulate TIL at a 2:1 aAPC to T cell ratio in the presence of exogenous IL-2 (100 IU/ml), or cultured in IL-2 containing medium alone. Representative results from one of three independent expansions are shown. After a single stimulation at a 2:1 aAPC to T cell ratio, TILs stimulated with anti-CD3 mAb loaded KT64/BBL aAPCs plus 100 IU/ml IL-2 expanded 100-fold over 9 days. In contrast, TILs did not undergo robust expansion when stimulated with KT32/BBL aAPCs when loaded with anti-CD3 mAb (6-fold); with anti-CD3/CD28 mAbs (6-fold); or with anti-CD3 mAb plus IL-2 (20-fold). These results show that robust TIL expansion is supported by single-round aAPC and IL-2 stimulation when the aAPCs express the high affinity Fc receptor CD64, but not CD32. Click here for file

Additional file 3 **Additional Figure S3**. PBLs and TILs from ovarian cancer patients have dissimilar differentiation phenotypes. TILs express lower levels of CD28 with an effector memory (CD45RO+ CD62L-) phenotype. **TILs outgrown from ovarian cancer specimens in IL-2 display a more differentiated phenotype compared to PBLs**. (**a**) Peripheral blood T lymphocytes express high levels of CD28 compared to T cells isolated from an autologous tumor explant. Histograms show CD28 surface expression by CD3-gated T cells from the blood (grey filled) or tumor (black filled) of the same patient with ovarian cancer. Isotype control is shown in empty gray line. (**b**) TILs outgrown in IL-2 preferentially display an effector memory (CD45RO+ CD62L-) skewed phenotype, relative to peripheral blood T cells from the same patient which exhibit diverse differentiation phenotypes including T central memory (CD45RO+ CD62L+) and naïve (CD45RO- CD62L+) cell phenotypes Click here for file

Additional file 4 **Additional Figure S4**. TILs expanded directly from enzyme-digested tumors are amenable to secondary expansion using aAPCs. **Young TILs expanded directly from fresh tumor digests are amenable to secondary expansion using aAPCs**. (**a**) 10^6 ^total tumor digest cells were stimulated with 10^6 ^aAPC loaded with anti-CD3 antibody with anti-CD28 agonist antibody in CM supplemented with 100 IU/mL IL-2. At day 9 of culture, aAPC stimulated TILs that had undergone modest primary expansion (185-fold mean) were re-stimulated using aAPC loaded with anti-CD3 antibody with anti-CD28 agonist antibody in CM supplemented with 100 IU/mL IL-2 for an additional 8 days. Mean viable cell ± SD counts are shown relative to day of stimulation (n = 3). (**b**) Fold expansion of CD3+ TILs. Pre- and post-expansion cells measured for contribution of viable CD3+ T cell contribution and used to calculate absolute T cell numbers (Total T cell number times % viable CD3+). Click here for file
