# Supplementary figures and images for: Engineered artificial antigen presenting cells facilitate direct and efficient expansion of tumor infiltrating lymphocytes
Source: J Transl Med. 2011 Aug 9;9:131. doi: 10.1186/1479-5876-9-131 (PMC3162913; doi:10.1186/1479-5876-9-131)

Fig. S1

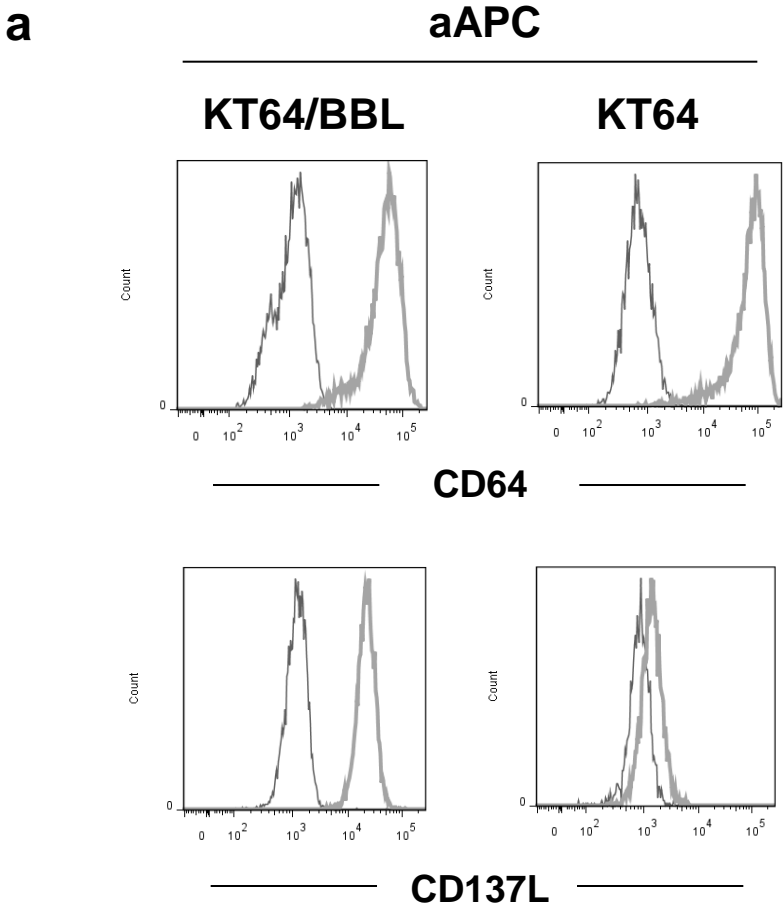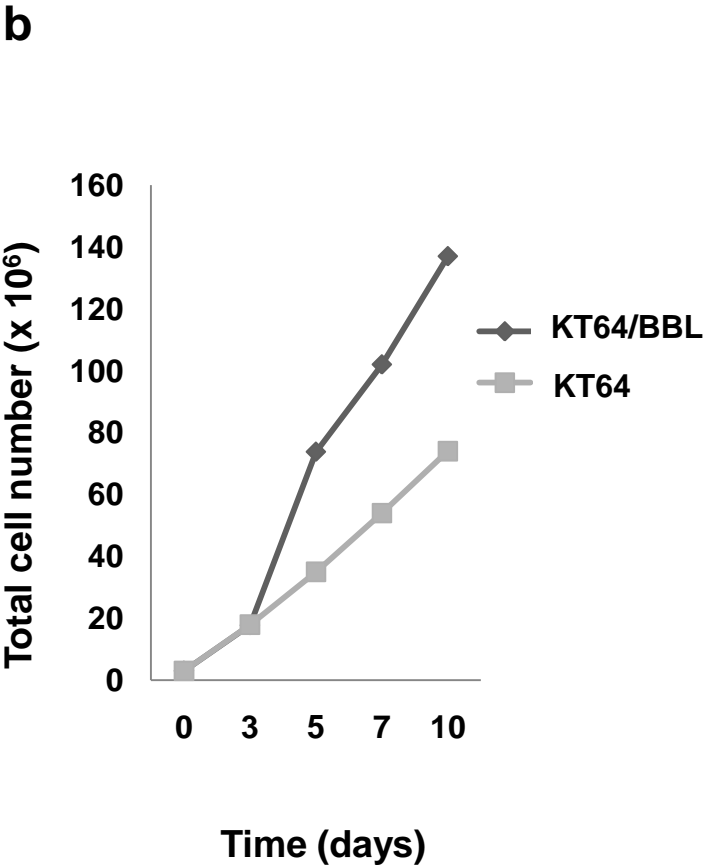

Supplement: Additional file 1 — Additional Figure S1. Characteristics of KT64/BBL aAPCs used for TIL expansion. 4-1BBL expression by the aAPC has a positive impact on TIL expansion potential. KT64/BBL aAPCs were generated to support the expansion of TILs. (a) aAPCs were genetically engineered with recombinant lentiviruses to express CD64 and CD137 (4-1BBL; referred to as KT64/BBL) or CD64 alone (KT64). Engineered cells were isolated by flow-sorting. Enriched KT64/BBL cells expressed high levels of CD64 and CD137L whereas KT64 expressed high levels of CD64 but not CD137L, as measured by flow cytometry. Specific antibodies are shown in gray; isotype antibody control is shown in black. (b) TIL expansion is augmented by CD137L stimulation. KT64/BBL aAPC pulsed with anti-CD3 antibody (0.5 ug/106 cells) and anti-CD28 antibody (0.5 ug/106 cells) stimulated enhanced TIL expansion at a 2:1 aAPC to T cell ratio in the presence of exogenous IL-2 (100 IU/ml), compared to KT64 control aAPC under identical conditions. [file 1479-5876-9-131-S1.PDF]

**Fig. S2**

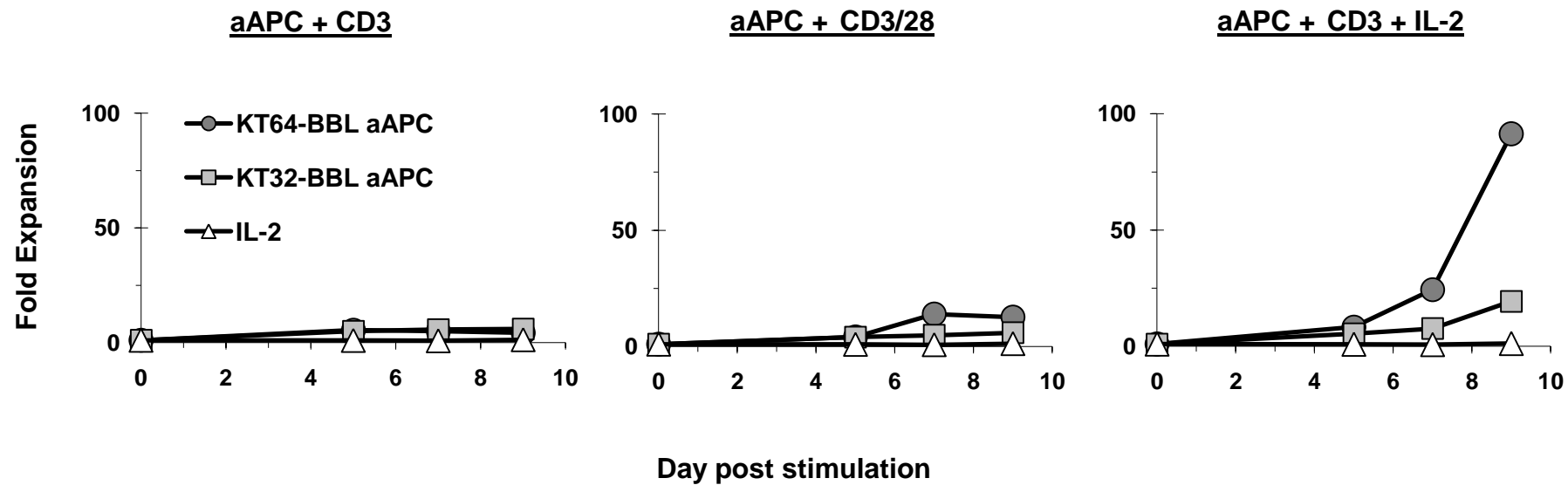

Supplement: Additional file 2 — Additional Figure S2. High affinity Fc gamma receptor CD64 is superior to the low affinity CD32 receptor for TIL expansion. K562 aAPC engineered to express CD64, but not CD32, induce rapid TIL expansion. K562 cells engineered to express 4-1BBL and the low affinity CD32/Fc-gammaRIII (KT32/BBL) or the high affinity CD64/FcgammaR1 receptor (KT64/BBL) were pulsed with anti-CD3 antibody (0.5 ug/106 cells) with or without anti-CD28 antibody (0.5 ug/106 cells) and used to stimulate TIL at a 2:1 aAPC to T cell ratio in the presence of exogenous IL-2 (100 IU/ml), or cultured in IL-2 containing medium alone. Representative results from one of three independent expansions are shown. After a single stimulation at a 2:1 aAPC to T cell ratio, TILs stimulated with anti-CD3 mAb loaded KT64/BBL aAPCs plus 100 IU/ml IL-2 expanded 100-fold over 9 days. In contrast, TILs did not undergo robust expansion when stimulated with KT32/BBL aAPCs when loaded with anti-CD3 mAb (6-fold); with anti-CD3/CD28 mAbs (6-fold); or with anti-CD3 mAb plus IL-2 (20-fold). These results show that robust TIL expansion is supported by single-round aAPC and IL-2 stimulation when the aAPCs express the high affinity Fc receptor CD64, but not CD32. [file 1479-5876-9-131-S2.PDF]

**Fig. S3**

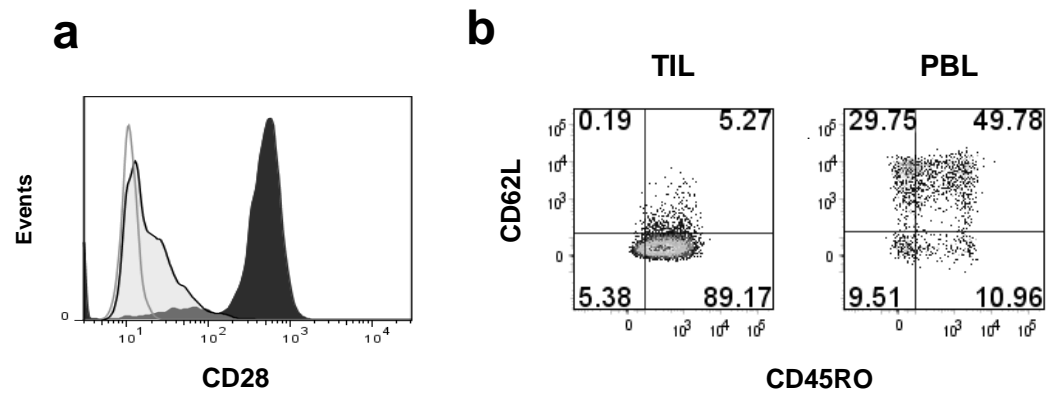

Supplement: Additional file 3 — Additional Figure S3. PBLs and TILs from ovarian cancer patients have dissimilar differentiation phenotypes. TILs express lower levels of CD28 with an effector memory (CD45RO+ CD62L-) phenotype. TILs outgrown from ovarian cancer specimens in IL-2 display a more differentiated phenotype compared to PBLs. (a) Peripheral blood T lymphocytes express high levels of CD28 compared to T cells isolated from an autologous tumor explant. Histograms show CD28 surface expression by CD3-gated T cells from the blood (grey filled) or tumor (black filled) of the same patient with ovarian cancer. Isotype control is shown in empty gray line. (b) TILs outgrown in IL-2 preferentially display an effector memory (CD45RO+ CD62L-) skewed phenotype, relative to peripheral blood T cells from the same patient which exhibit diverse differentiation phenotypes including T central memory (CD45RO+ CD62L+) and naïve (CD45RO- CD62L+) cell phenotypes [file 1479-5876-9-131-S3.PDF]

**Fig. S4**

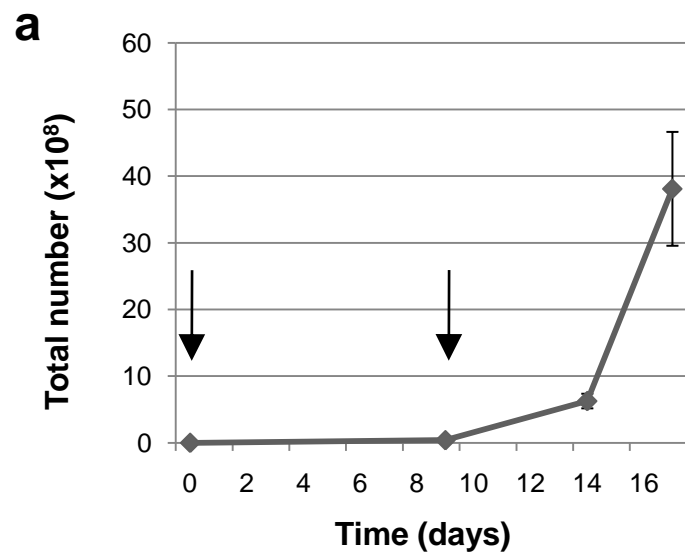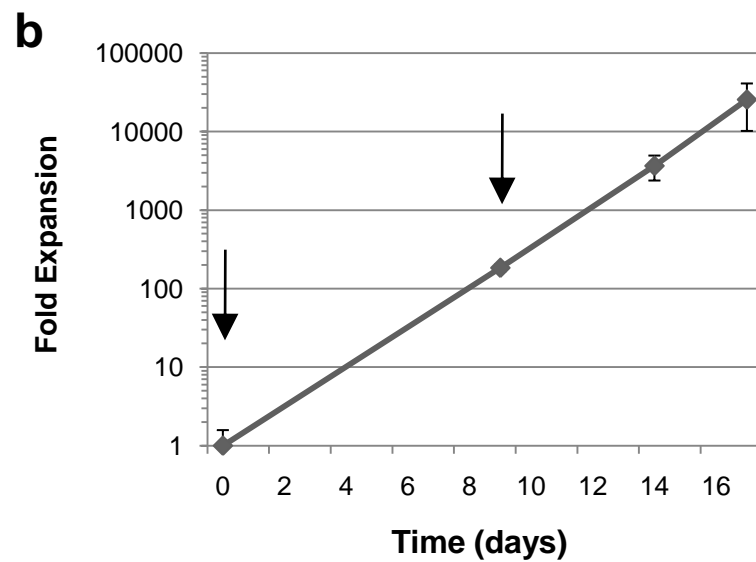

Supplement: Additional file 4 — Additional Figure S4. TILs expanded directly from enzyme-digested tumors are amenable to secondary expansion using aAPCs. Young TILs expanded directly from fresh tumor digests are amenable to secondary expansion using aAPCs. (a) 106 total tumor digest cells were stimulated with 106 aAPC loaded with anti-CD3 antibody with anti-CD28 agonist antibody in CM supplemented with 100 IU/mL IL-2. At day 9 of culture, aAPC stimulated TILs that had undergone modest primary expansion (185-fold mean) were re-stimulated using aAPC loaded with anti-CD3 antibody with anti-CD28 agonist antibody in CM supplemented with 100 IU/mL IL-2 for an additional 8 days. Mean viable cell ± SD counts are shown relative to day of stimulation (n = 3). (b) Fold expansion of CD3+ TILs. Pre- and post-expansion cells measured for contribution of viable CD3+ T cell contribution and used to calculate absolute T cell numbers (Total T cell number times % viable CD3+). [file 1479-5876-9-131-S4.PDF]
